# Clinical Outcomes of BCMA CAR-T Cells in a Multiple Myeloma Patient With Central Nervous System Invasion

**DOI:** 10.3389/fonc.2022.854448

**Published:** 2022-05-16

**Authors:** Ting Wang, Ting He, Lie Ma, Yazi Yang, Ru Feng, Yanping Ding, Yueming Shan, Bing Bu, Feifei Qi, Fei Wu, Xin-an Lu, Hui Liu

**Affiliations:** ^1^ Department of Hematology, Beijing Hospital; National Center of Gerontology; Institute of Geriatric Medicine, Chinese Academy of Medical Science, Beijing, China; ^2^ Immunochina Pharmaceuticals Co., Ltd., Beijing, China; ^3^ China Medical University, Shenyang, China; ^4^ Shandong Academy of Medical Sciences, Jinan, China

**Keywords:** CAR-T, BCMA, multiple myeloma, central nervous system, case report

## Abstract

**Background:**

Multiple myeloma (MM) is the second most common hematological malignancy that still lacks effective clinical treatments. In particular, MM with central nervous system (CNS) invasion occurs rarely. Although B-cell maturation antigen (BCMA)-targeted chimeric antigen receptor-T (CAR-T) cell therapy has shown great promise for the treatment of relapsed/refractory MM, few studies have reported whether BCMA CAR-T could inhibit MM with CNS invasion.

**Case Presentation:**

In this study, we report a special case of a 63-year-old male patient who suffered MM with CNS invasion and presented rapid extramedullary disease (EMD) progression into multiple organs. Before CAR-T cell infusion, this patient received five cycles of bortezomib, Adriamycin, and dexamethasone (PAD) and an autologous transplant as the front-line treatment, followed by two cycles of bortezomib, lenalidomide, and dexamethasone (VRD) as the second-line regimen, and daratumumab, bortezomib, dexamethasone (DVD) as the third-line regimen. Since the patient still showed rapid progressive disease (PD), BCMA CAR-T cells were infused, and 1 month later, a stringent complete response (sCR) was achieved, and the response lasted for 4 months. Meanwhile, only grade 1 cytokine release syndrome (CRS) was observed.

**Conclusion:**

This case report demonstrated that BCMA CAR-T could effectively eradicate CNS-involved MM with low adverse events, suggesting that CAR-T cell therapy could be a feasible therapeutic option for this kind of refractory disease.

**Clinical Trial Registration:**

https://ClinicalTrials.gov, identifier: NCT04537442.a

## Introduction

Multiple myeloma (MM) is a malignancy derived from terminally differentiated plasma cells and has become the second most prevalent hematological cancer (1). Extramedullary disease (EMD) sometimes occurs in the late-stage MM and is present in about 5% of MM patients. The incidence of EMD in the central nervous system (CNS) is even rare and is reduced to <1% of MM patients (2). MM with CNS invasion (CNS-MM) could be triggered by various pathological factors and is an intractable complication (3). Multiple treatments including proteosome inhibitors, immunomodulatory drugs, local radiation, and autologous stem cell transplantation have been applied in clinical therapy. However, durable response is difficult to achieve, and the median survival of CNS-MM patients is only 3 months (3, 4). Thus, additional therapeutic strategies for CNS-MM need to be developed.

Chimeric antigen receptor-T (CAR-T) therapy has shown great promise to control relapsed/refractory MM, particularly B-cell maturation antigen (BCMA)-targeted CAR-T cells. The Food and Drug Administration (FDA) has approved two BCMA CAR-T therapies, including BMS’s Abecma (idecabtagene vicleucel, ide-cel), and Carvykti (ciltacabtagene autoleucel, cilta-cel) by Janssen and Legend Biotech (5–7). The overall response rate (ORR) of these products was reported to reach 73%–97%, and the median progression-free survival time achieved over 8.8 months (6–8). However, few studies have investigated whether BCMA CAR-T could penetrate the blood–brain barrier and eradicate the CNS-MM tumors. Recently, Wang et al. retrospectively analyzed four cases of CNS-MM patients who received BCMA CAR-T therapy and found that three cases showed complete response (CR) with grades 1–2 cytokine release syndrome (CRS) and no neurotoxicity, suggesting the safety and feasibility of BCMA CAR-T to treat CNS-MM (9).

To support the clinical application of CAR-T therapy for CNS-MM, here, we reported a case study of BCMA CAR-T in an MM patient with severe cytogenetic abnormalities and multiple extramedullary infiltration sites including CNS invasion. After a single infusion of BCMA CAR-T cells, the patient achieved a stringent complete response (sCR) within 1 month and showed durable response for 4 months. The encouraging result demonstrated that BCMA CAR-T therapy is an optimal strategy for the treatment of severe CNS-MM and is worthy of in-depth investigation in a large cohort of patients in future.

## Case Presentation

### Treatment History of Patient

A 63-year-old male patient presented to our hospital in July 2018. The patient had a symptom of coughing and right-sided rib pain. CT scans showed mass in thoracic spine 6–8, vertebral fracture, and left pleural effusion. This patient was diagnosed as IgA-λ MM at the DS IIIA stage (or ISS III, R-ISS III stage with EMD), exhibiting 67% and 10.48% of myeloma cells from morphology and immunophenotyping analysis, respectively. In addition, cytogenetic analysis of bone marrow by direct fluorescence *in situ* hybridization (D-FISH) panels was performed. There were 53% 1q21 amplification, 7.5% RB1 13q14 loss, 67.5% D13S19 13q14.3 loss, 7% IgH 14q32 recombination, and 4% p53 17q13 loss. PET-CT scans of the patient showed (1) diffuse increase in bone marrow metabolism, accompanying largely osteolytic change and left anterior second rib pathological fracture; (2) T6–9 vertebral left-sided soft tissue mass with enhanced metabolism; and (3) hypermetabolic and enlarged mediastinal lymph node in zone 7. No other genetic information from his family was revealed.

Before CAR-T cell infusion, three lines of therapy were implemented for this patient. For the front-line therapy, the patient received five cycles of PAD chemotherapy, which consisted of bortezomib, doxorubicin, and dexamethasone. It resulted in partial response (PR) after the first cycle, very good partial response (VGPR) after the second cycle, and VGPR after the fourth cycle. After that, plerixafor was used to mobilize the hematopoietic stem cell (HSC) for autologous HSC transplant (ASCT). High-dose melphalan (200 mg/m^2^ of body surface area) was used for preconditioning. ASCT was performed successfully, and sCR was achieved 1 month after transplantation. Maintenance therapy was applied using bortezomib and dexamethasone (BD) regimen every 3 months as one cycle and daily treatment of 100 mg thalidomide between cycles. However, the myeloma relapsed 4 months after the transplant. For the second-line therapy, the patient received two cycles of VRD (bortezomib, lenalidomide, and dexamethasone) chemotherapy. Unfortunately, the patient showed progressive disease (PD) after two cycles. A cycle of DVD (daratumumab, bortezomib, and dexamethasone) chemotherapy regimen was then used as a third-line therapy, but the response was still evaluated as PD. The blood examination showed 4,090 mg/dl of serum IgA, 645 U/L of lactic dehydrogenase (LDH), 34 g/L of monoclonal protein, and 19% of myeloma cells in the bone marrow. Based on IgH/CCND t (11; 14) by the FISH examination of myeloma cells after CD138-positive cell sorting, venetoclax was added into the DVD regimen. However, disease progression was not alleviated. At that time, the patient presented the severe symptoms such as pancytopenia, neutrophil deficiency (0.15×10^9^/L), reduced platelets (4×10^9^/L), bleeding gingival, massive bloody sputum, skin ecchymosis, muscle weakness in both lower limbs (grades 2–3), numbness, difficulty in to standing, abnormal feelings in hips, urinary retention, obvious pain in the whole body (VAS 10), and diplopia. The blood coagulation parameters were normal. Computed tomography (CT) scans showed multiple bone destruction, low-density areas in the liver and spleen, and multiple abdominal nodules, while magnetic resonance imaging (MRI) examination of the head detected abnormal signals in cauda equina and the right parietal lobe region that was close to falx cerebri. Orbital MRI was not performed because the patient had severe symptoms of pain and could not be relieved using drugs. Ophthalmic examination of the patient showed that there was no abnormality in intraocular pressure, fundus and visual acuity, and optic papilla edema. However, the patient was sensitive to light reflection and had double vision, slight limitation of right eye abduction, and defective monocular vision, and no nystagmus. The neurological examination exhibited symmetrical bilateral facial pain, symmetrical facial lines, and normal mouth angle of teeth and the tongue position. The muscle strength was grade 4–5 for both upper limbs, grade 2 for the left lower limbs, and grade 2–3 for the right lower limbs. Based on the symptoms and imaging characteristics, the patient was diagnosed with relapsed/refractory MM with CNS invasion seriously at this time. In addition to the CNS, the liver, spleen, and skin were also invaded with tumors.

### Treatment With BCMA CAR-T Cells

**Figure 1:**
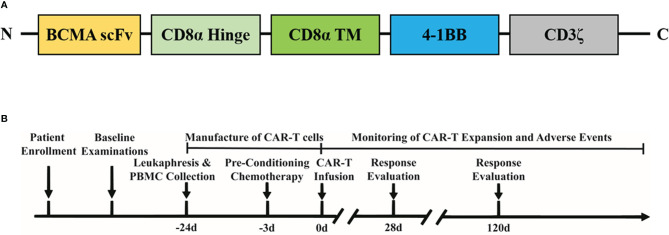
Schematic illustration of the CAR construct and treatment timeline. **(A)** Composition of CAR molecule. **(B)** Clinical study design.

In October 2019, the patient was enrolled into the BCMA CAR-T (IM21 CAR-T, autologous anti-BCMA CAR-T cells that recognize and induce selective toxicity against BCMA-expressing tumor cells) clinical trial (https://ClinicalTrials.gov identifier: NCT04537442). This study was conducted in accordance with the Declaration of Helsinki and was approved by the Institutional Review Board at Beijing Hospital in China. The patient was informed of the possible risks and side effects of the therapy and signed informed consent. No bridging chemotherapy, radiation therapy, or intra-thecal treatment was used before CAR-T cell treatment, as the patient showed a very low level of platelet (4×10^9^/L) and symptoms of severe pain. The patient received lymphodepletion treatment with fludarabine (25 mg/m^2^/day) and cyclophosphamide (250 mg/m^2^/day) for 3 consecutive days before CAR-T infusion. BCMA CAR-T cells were prepared using the good manufacturing practice (GMP) facilities at Immunochina Pharmaceuticals (Beijing, China). The detailed method of CAR-T preparation was as previously described (10). The schematic structure of BCMA CAR construct is shown in **Figure 1A**, using a murine scFv against BCMA, CD8α hinge and transmembrane region, 4-1BB co-stimulatory domain, together with CD3ζ signaling domain. The BCMA CAR gene was transduced to activated T cells using lentiviral vectors. When the CAR-T cells expanded to sufficient dose, cells were cryopreserved and underwent quality-control evaluations. The viability of BCMA CAR-T cells was 88%, and the CAR expression ratio was 34.32%. The CD4/CD8 ratio of CAR-T cells was approximately 0.82. About 30% of CAR-T cells were central memory T cells (CD45RA^−^CD62L^+^), 65% were naive and stem cell memory T cells (CD45RA^+^CD62L^+^), 2.3% were effector memory T cells (CD45RA^−^CD62L^−^), and 2.7% were effector T cells (CD45RA^+^CD62L^−^) based on flow cytometry analysis shown in **Supplementary Figure S1**. On day 0, the patient was infused with BCMA CAR-T cells (1×10^6^/kg, 6.6×10^7^ in total). Adverse events were evaluated according to the Common Terminology Criteria for Adverse Events (CTCAE) Version 5.0. The severity of cytokine release syndrome (CRS) and neurotoxicity was assessed according to the reported gradings. The primary endpoint was incidence of adverse events within 1 month after CAR-T cell infusion. The secondary endpoint was objective response rate at 3 months. Patients were followed up at days 0, 4, 7, 10, 14, 21, 28, 90, and 180, and 1 year. A schematic design of the clinical trial is shown in **Figure 1B**.

### Response to Treatment

**Figure 2:**
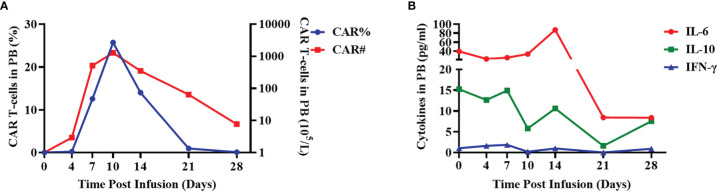
Evaluation of CAR ratio, the number of CAR-T cells and cytokines in the peripheral blood. **(A)** The ratio of BCMA CAR-T cells in total lymphocytes (CAR%) and the absolute number of BCMA CAR-T cells (CAR#) in the peripheral blood of the patient were detected using flow cytometry at the indicated days after CAR-T infusion. **(B)** The expression levels of cytokines (IL-6, IL-10, and IFN-γ) in the peripheral blood were evaluated using cytometric bead array after CAR-T infusion at the indicated time points.

The patient was closely followed up within 1 month after CAR-T cell infusion. The peripheral blood was collected at days 0, 4, 7, 10, 14, 21, and 28 and was analyzed using flow cytometry to measure the CAR-T ratio, CAR-T cell number, and cytokine levels. CAR-T cells expanded and reached the maximal level in peripheral blood on day 7 post-infusion (**Figure 2A**). Both serum interleukin (IL)-10 and interferon gamma (IFN-γ) reached the peak level on day 7 (14.9 and 1.8 pg/mL, respectively), and the level of IL-6 was highest on day 14 (87.4 pg/mL, **Figure 2B**). In accordance with the cytokine expression, the patient experienced grade 1 CRS and developed symptoms of fever (Tmax, 39.2°C) and headache on day 7. The Visual Analogue Scale score was evaluated as 6. The blood pressure was 129/75 mmHg, and the oxygen saturation was 99%. The level of ferritin was 2,983 ng/ml, and the level of C-reactive protein was 3.5 mg/dl in the peripheral blood on day 7. Blood routine analysis showed the level of leukocyte as 4.97×10^9^/L, and the level of neutrophil was 4.15×10^9^/L. Other symptoms including nausea, vomit, cough, diarrhea, and chill were not observed. Levetiracetam, dexamethasone, and mannitol were used to relieve the CRS symptoms. On day 9, the temperature decreased to normal level, and headache was relieved. The patient showed clear consciousness, accurate sense of orientation, accurate answering of questions, and no symptoms of tremor, dyslexia, aphasia, epilepsy, and neck rigidity after CAR-T therapy. Thus, no neurotoxicity was observed in this patient.

**Figure 3:**
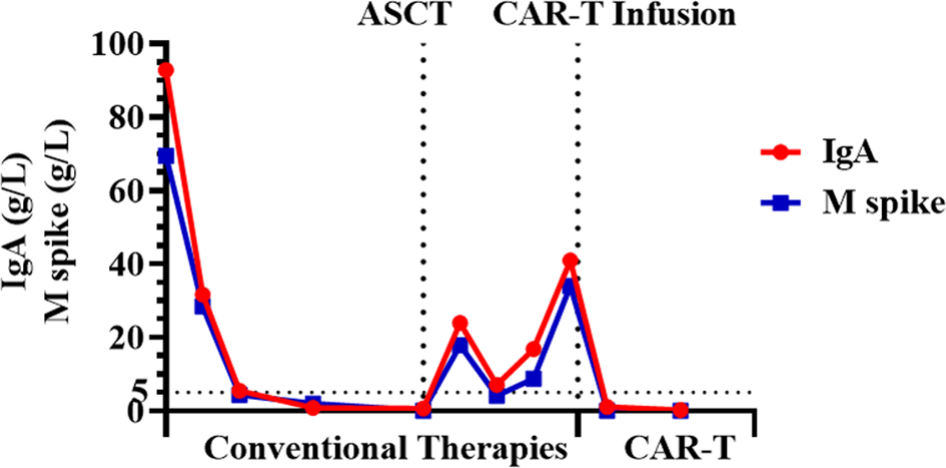
The concentrations of IgA and M spike before and after BCMA CAR-T cell infusion.

**Figure 4:**
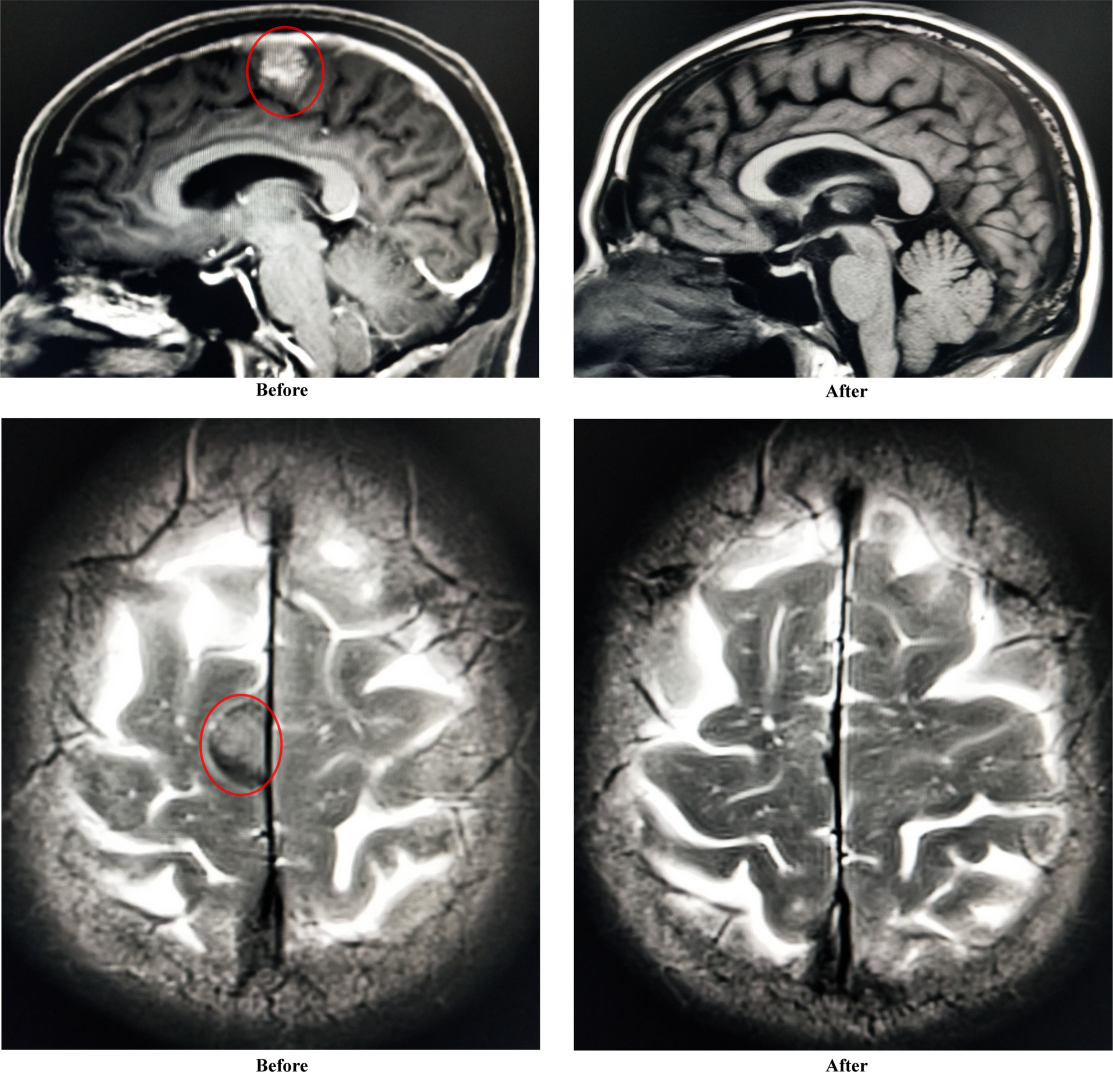
Improvement of abnormal signals in right parietal lobe region (sagittal plane and transverse plane) before and after CAR-T infusion.

One month post-infusion, the patient suffered no pain and no bloody sputum. The lower limb muscle weakness was improved (4 to 4+ grade), and diplopia was turning better. One month post-CAR-T infusion, the level serum IgA was very low, and monoclonal protein (M-spike) turned to be negative. The level of M-spike remained negative even at 3 months (**Figure 3**). The concentrations of free κ and λ chains in serum were 6.1 and 5.29 mg/L, respectively, with a κ/λ ratio of 1.15. Bone marrow aspiration sample showed no abnormal plasma cells from morphology observation, and only 0.06% of myeloma cells were detected using immunophenotyping analysis. More importantly, the CNS-involved tumors were eradicated. Cranial and orbital MRI showed no abnormalities, and previous abnormal signals in the right parietal lobe region disappeared (**Figure 4**). Additionally, other EMD lesions including those in the skin, liver, and spleen also disappeared. Together, the patient response was evaluated as sCR 1 month after CAR-T infusion.

Four months later, the situation was much better improved, and the response remained sCR based on the following evidence. The patient experienced neither pain nor any hemorrhage. The lower limb muscle strength became normal, and diplopia was much more alleviated. Myeloma cells further reduced to 0.02% from immunophenotyping analysis. M-spike still remained negative, and the levels of free κ and λ chains in the serum were 5.5 and 26.9 mg/L, with a κ/λ ratio of 0.20. No lesions with increased metabolic activity were observed through whole-body PET-CT imaging.

## Discussion

CNS-MM is a rare complication, since only 10 CNS-involved cases could be identified in a total of 5,238 MM patients as reported by Leeds Cancer Center in UK (2). Due to the heterogeneous symptoms, the diagnosis of this infrequent disease was intricate, and patients generally showed very poor prognosis. At present, effective clinical regimens for CNS-MM are still scanty, and novel therapies need to be investigated to fill the gap. This study reported an efficient and safe BCMA CAR-T cell therapy for a CNS-MM patient with multiple severe extramedullary lesions. One month post-infusion of CAR-T cells, the patient was evaluated as response of sCR and very low grade of adverse effects. Compared to the traditional regimens, BCMA CAR-T therapy exhibited great potential to eradicate MM lesions and suppress the progression of disease even at the relapsed/refractory stage and with CNS invasion.

In the past 5 years, CAR-T therapy has been largely explored for the treatment of MM patients in clinical studies. The satisfactory expression pattern of BCMA in MM tumors and normal tissues enabled it as a suitable target for CAR-T development. Based on published data from multiple clinical trials, BCMA CAR-T therapy exhibited good safety and initial clinical response in most relapsed/refractory MM patients (11, 12). Ide-cel was reported to show 73% of overall response rate, 33% of complete response rate, and a median progression free survival time of 8.8 months in a cohort of 128 patients (6). A phase 1b/2 study of Cilta-cel reported more amazing data and showed 97% of overall response rate, 67% of stringent complete response, and 77% of 12-month progression-free survival rate (7). These investigations suggested a rapid and broad initial response of MM patients to BCMA CAR-T therapy; however, tumor relapse still occurred in a proportion of patients. Although factors correlated with the durable response of BCMA CAR-T therapy have not been well-defined, the presence of EMD in MM, especially the complicated CNS invasion, was a possible attribute for the drug resistance and tumor recurrence. The coming challenges for BCMA CAR-T therapy will focus on elongation of durable response time and inhibition of tumor relapse (11, 13). Extensive investigations are required in areas including analysis of efficacy-associated biomarkers, optimization of CAR-T molecular design and manufacturing technology, combination therapy, and clinical exploration of CAR-T therapy in special MM types such as CNS-MM. In our case study, the patient previously received three lines of therapies but still suffered from rapid disease progression and severe EMD. Facing a stern situation, the patient attempted to receive the BCMA CAR-T therapy, and a remarkable clinical response was achieved within 1 month. The response lasted for 5 months, and then, tumor relapse occurred outside the central nervous system. The patient received additional regimens to control the relapsed tumors, including Bendamustine and BCL-2 inhibitors. Unfortunately, the patient died at 1 year after CAR-T treatment. This was our first investigation of BCMA CAR-T therapy for CNS-MM patients. In accordance with our findings, two recent retrospective clinical studies by Wang et al. demonstrated the favorable clinical outcomes of BCMA CAR-T cells in several CNS-MM patients (9, 14). The structures of CAR molecules in these reports and our study were similar, as the second-generation CAR with murine scFv and 4-1BB co-stimulatory domain was utilized. However, the manufacturing process was probably different. Meanwhile, the case in our study showed more severe cytogenetic abnormalities and extramedullary lesions in multiple organs. Another study by Zhang et al. also reported the application of BCMA CAR-T in a CNS-MM patient (15). This study evaluated a BCMA CAR-T with CD28 and OX40 co-stimulatory domain that was different from our CAR-T structure. Meanwhile, partial response and grade 4 neurotoxicity was observed in the patient. The distinct clinical response and adverse events between this case report and our study were possibly attributed to the different CAR structure and manufacturing process. Our study together with previous reports supported the clinical application of BCMA CAR-T cells for CNS-MM patients even with severe complications. Nevertheless, in-depth clinical investigations are required to elucidate the safety and efficacy of BCMA CAR-T cells in a larger cohort of CNS-MM patients.

A key question then arises: can CAR-T cells infiltrate the blood–brain barrier (BBB) and be functional in the central nervous system? Direct evidence cannot be obtained from previous studies of CAR-T therapy in MM. Nevertheless, reports of CD19 CAR-T therapy in B-cell acute lymphoblastic leukemia (B-ALL) and diffuse large B-cell lymphoma (DLBCL) provided certain hints. The permeability of BBB increased among patients receiving CD19 CAR-T therapy and permitted CAR-T cells to infiltrate into the brain from blood circulation (16). In line with this, another study reported the expression of CD19 in a population of brain mural cells surrounding the blood vessels, leading to the on-target-off-tumor effect of CD19 CAR-T cells and BBB leakiness (17). Based on the capacity of CAR-T cells to infiltrate into the central nervous system, several studies investigated the effects of CD19 CAR-T cells on CNS-involved malignancies. Frigault et al. reported the clinical outcomes of Tisagenlecleucel in eight DLBCL patients with secondary CNS tumors and found that four patients showed effective responses. No patient experienced >grade 1 neurotoxicity, indicating that the interaction of CAR-T cells with tumor cells in the central nervous system could not induce severe adverse events (18). Other studies also showed that aggressive DLBCL and B-ALL patients with CNS involvement could achieve long-term tumor remission and manageable adverse events following CD19 CAR-T treatment (19, 20). These studies support that CAR-T cells can penetrate the BBB and effectively eliminate the CNS tumor lesions. A disadvantage of our study lies in that the detection of BCMA CAR-T cells in the cerebrospinal fluid was not successfully performed. After CAR-T infusion, lumbar puncture could not be carried out, as the patient showed an extremely low level of platelet (4×10^9^/L). Future studies of BCMA CAR-T cell level and cytokine expression patterns in the cerebrospinal fluid will be beneficial to the understanding of CAR-T therapy for CNS-MM.

Taken together, our case study demonstrated the effectiveness and safety of BCMA CAR-T in a CNS-MM patient with multiple other extramedullary lesions. Thus, CAR-T cell therapy has a potential as a candidate treatment for patients with CNS-MM. Further prospective studies with broader patient populations and longer follow-up periods are still required to validate this conclusion.

## Data Availability Statement

The original contributions presented in the study are included in the article. Further inquiries can be directed to the corresponding authors.

## Ethics Statement

The studies involving human participants were reviewed and approved by Institutional Review Board at Beijing Hospital. The patients/participants provided their written informed consent to participate in this study. Written informed consent was obtained from the individual(s) for the publication of any potentially identifiable images or data included in this article.

## Author Contributions

TW, TH, LM, FW and HL conceived and designed the study. TW, YY, RF and HL performed clinical examinations. TW, TH, YD, YS, BB, FQ, FW, XL and HL analyzed and interpreted the data. TH, YD, FQ and XL designed the CAR and prepared the CAR-T cell product. TW, YD and YS wrote the manuscript. All authors contributed to the article and approved the submitted version.

## Funding

This study was financially supported by Beijing Municipal Science & Technology Commission (Z181100006218036, Z181100002218040, and Z191100001119097) and Capital Clinical Characteristic Application Research (Z181100001718162).

## Conflict of Interest

TH, YD, YS, FQ, FW, and XL are employed by the Immunochina Pharmaceuticals Co. Ltd.

The remaining authors declare that the research was conducted in the absence of any commercial or financial relationships that could be construed as a potential conflict of interest.

## Publisher’s Note

All claims expressed in this article are solely those of the authors and do not necessarily represent those of their affiliated organizations, or those of the publisher, the editors and the reviewers. Any product that may be evaluated in this article, or claim that may be made by its manufacturer, is not guaranteed or endorsed by the publisher.

## Abbreviations

MM, multiple myeloma; CNS, central nervous system; BCMA, B-cell maturation antigen; CAR-T, chimeric antigen receptor-modified T cell; sCR, stringent complete response; PR, partial response; PD, progressive disease; VGPR, very good partial response; CRS, cytokine release syndrome; EMD, extramedullary disease; ORR, overall response rate; FISH, fluorescence *in situ* hybridization; HSC, hematopoietic stem cell; ASCT, autologous HSC transplant; MRI, magnetic resonance imaging.
