# Supplementary material for: Clinical Outcomes of BCMA CAR-T Cells in a Multiple Myeloma Patient With Central Nervous System Invasion
Source: Front Oncol. 2022 May 16;12:854448. doi: 10.3389/fonc.2022.854448 (PMC9150173; doi:10.3389/fonc.2022.854448)
Supplement: Supplementary file 1 [file DataSheet_1.pdf]

Clinical Outcomes of BCMA CAR-T Cells in a Multiple Myeloma Patient With Central Nervous System Invasion

Ting Wang[1†], Ting He[2†], Lie Ma[3†], Yazi Yang[1], Ru Feng[1], Yanping Ding[2], Yueming Shan[2], Bing Bu[4], Feifei Qi[2], Fei Wu[2], Xin-an Lu[2*], Hui Liu[1*]

[1]Department of Hematology, Beijing Hospital; National Center of Gerontology; Institute of Geriatric Medicine, Chinese Academy of Medical Science, Beijing 100730, China
[2]Immunochina Pharmaceuticals Co., Ltd, Beijing 100089, China
[3]China Medical University, Shenyang 110122, Liaoning, China
[4]Shandong Academy of Medical Sciences, Jinan 250022, Shandong, China

***Correspondence:**
Xin-an Lu
luxinan@immunochina.com
Hui Liu
liuhui8140@126.com;

[†]These authors have contributed equally to this work and share first authorship

Figure S1

A

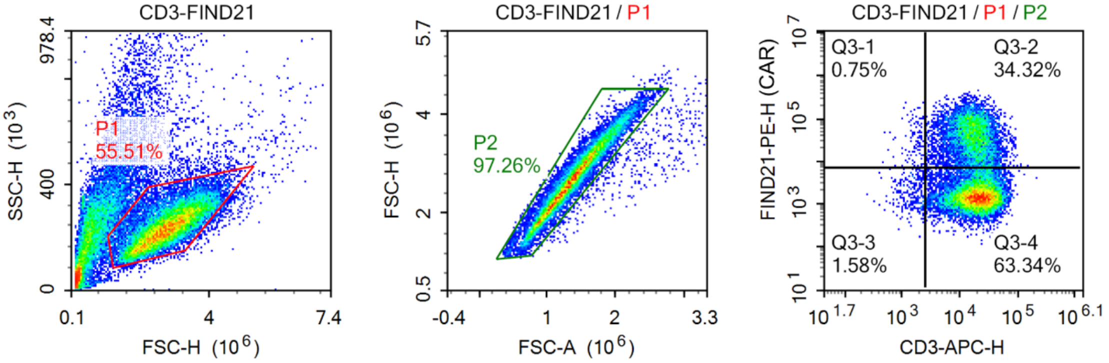

B

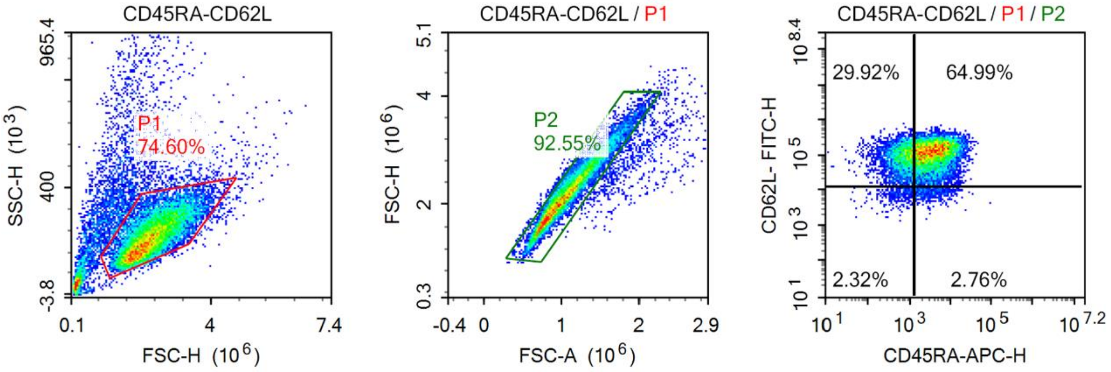

C

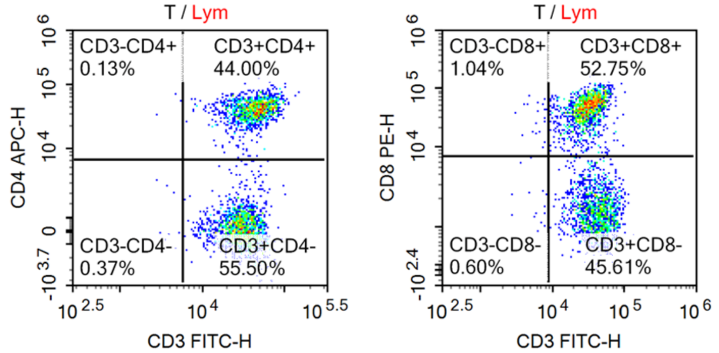

Figure S1. The CAR expression ratio **(A)**, different subsets of T cells **(B)** and CD4/CD8 ratio **(C)**.
